# Phytochemical Profile, Antioxidant Activity, and Neuroprotective Effects of *Bacopa monnieri* Extract in a Lipopolysaccharide-Induced Dementia Model

**DOI:** 10.3390/ijms27125229

**Published:** 2026-06-09

**Authors:** Abosede Temitope Olajide, Sasithon Aunsorn, Samuel Abiodun Kehinde, Shang Yazhen, Thammarat Kaewmanee, Sasitorn Chusri

**Affiliations:** 1 Cell and Signaling Laboratory, Department of Biomedical Science, Faculty of Medicine and Health Sciences, Universiti Putra Malaysia (UPM), Seri Kembangan 43400, Malaysia; abosedeolajide3@gmail.com; 2 Biomedical Technology Research Group for Vulnerable Populations, School of Health Science, Mae Fah Luang University, Chiang Rai 57100, Thailand; samuelkehinde0707@gmail.com; 3 Department of Thai Traditional Medicine, Faculty of Allied Health Sciences, Nakhon Ratchasima College, Nakhon Ratchasima 30000, Thailand; 6551811005@lamduan.mfu.ac.th; 4 Biochemical/EnTox Laboratory, Faculty of Basic Medical Sciences, Ajayi Crowther University, Oyo Town 211001, Oyo State, Nigeria; 5 Institute of Traditional Chinese Medicine, Chengde Medical University, Chengde 067000, China; shangyz1018@hotmail.com; 6 Department of Food Science and Nutrition, Faculty of Science and Technology, Prince of Songkla University, Pattani 94000, Thailand; thammarat.k@psu.ac.th

**Keywords:** antioxidant, *Bacopa monnieri*, cognitive function, lipopolysaccharide-induced dementia, neuroprotection

## Abstract

*Bacopa monnieri* (BM) is a traditional medicinal herb that has been reported to have neuroprotective and cognitive-enhancing properties. In this study, the antioxidant, safety, and neuroprotective properties of BM extract (BME) were assessed in a lipopolysaccharide (LPS) model of cognitive impairment. Ethanol was used for extraction, after which the ethanolic extract was profiled to characterize total phenolic and flavonoid content and major bioactive constituents. The assessment of antioxidant activity was done through several in vitro tests (DPPH, ABTS, FRAP, NBT, OARC, and metal chelation). Toxicity was assessed in *Caenorhabditis elegans* using pharyngeal pumping and food clearance tests. For in vivo evaluation, rats were pre-treated with BME, and then LPS was administered, followed by evaluation of cognitive performance by the Morris water maze and Y-maze test. Phytochemical examination revealed the existence of phenolics and flavonoids, as well as bacoside A components. The extract showed good antioxidant activity, mainly via hydrogen atom transfer and single-electron transfer, suggesting effective radical scavenging and reducing ability, but no metal chelating activity was observed. Toxicity tests demonstrated that lower concentrations of the extract were well tolerated, and higher concentrations resulted in temporary inhibition of feeding behavior, indicating mild, dose-dependent effects. In the LPS-induced rat model, the inflammatory challenge produced significant cognitive deficits relative to normal controls, validating the model. Pre-treatment with BME at 70 mg/kg did not produce statistically significant rescue of any behavioral endpoint compared with the LPS-only group, although small-to-medium effect sizes in the protective direction were observed for several measures. Additionally, BME modulated LPS-induced neuroinflammatory responses by reducing cortical IL-1β, TNF-α, iNOS, and COX-2 levels while enhancing hippocampal AChE and PGE2 activity, suggesting region-specific anti-inflammatory and cholinergic regulatory effects. The most robust positive findings of this study are therefore the phytochemical characterization and the in vitro antioxidant profile of this standardized extract, which support its potential as a candidate for further investigation in inflammation-related cognitive impairment; the in vivo findings are preliminary and warrant confirmation in larger-scale, dose-ranging studies.

## 1. Introduction

Dementia is a neurodegenerative syndrome, a progressive condition characterized by memory loss as well as other cognitive impairments that significantly affect daily activities and quality of life [1]. Dementia affects approximately 57 million individuals worldwide today and is likely to increase exponentially as the population ages. The most prevalent type is Alzheimer’s disease (AD), accounting for approximately 60–70% of cases [2]. The burden is increasing in aging populations, particularly in the Asia-Pacific region, which is predicted to be the major source of dementia cases worldwide by 2050 [3]. The social and economic costs are immense; dementia care has been estimated to cost around USD 1.3 trillion annually worldwide, reflecting the burden on health care systems and caregivers [4].

Dementia pathogenesis is a complex, interrelated process. The buildup of misfolded proteins (amyloid-β plaques and hyperphosphorylated tau tangles), mitochondrial dysfunction, and oxidative stress induce chronic neuroinflammation [5]. Activated microglia and astrocytes release pro-inflammatory cytokines (e.g., tumor necrosis factor-alpha (TNF-α), interleukin-6 (IL-6)) and reactive oxygen species, creating a vicious cycle of neuronal damage [6,7,8]. New evidence shows that the gut–brain axis is a crucial modulator: intestinal dysbiosis and increased gut permeability may allow bacterial components (including lipopolysaccharide, LPS) and other metabolites to enter the circulation, triggering systemic inflammation and microglial activation in the brain. By so doing, peripheral inflammation and microbiota imbalance could increase central neurodegeneration and cognitive decline [9].

Dementia has no cure, although current treatment options only control the symptoms and delay the progression. Cholinesterase inhibitors (donepezil, rivastigmine, galantamine) and the *N*-methyl-D-aspartate (NMDA) receptor antagonist memantine remain the primary pharmacological interventions for the treatment of Alzheimer’s disease, offering only a modest level of symptomatic improvement and not delaying disease progression [10]. Recently licensed anti-amyloid monoclonal antibodies (e.g., lecanemab, donanemab) can modestly reduce cognitive decline in the early stages of AD, but their effects are limited by access issues and side effects [11]. Simultaneously, the benefits of non-pharmacological interventions, such as aerobic training, Mediterranean-style nutrition, cognitive-behavioral training, and sleep hygiene, have been proven in terms of memory, attention, and executive function [12]. Nonetheless, the net effect of existing treatment methods remains limited, and numerous dementia medications have side effects, which are the reason to raise the interest in such complementary and alternative therapy. Namely, the potential of natural products containing a high content of bioactive phytochemicals (flavonoids, terpenoids, phenolics, etc.) as neuroprotective antioxidants and anti-inflammatory agents is under investigation and could affect various disease pathways in dementia [13,14].

In Ayurvedic medicine, *Bacopa monnieri* (also known as Brahmi) is a widely known herbal medicine and a Medhya Rasayana (memory-enhancing) plant [15,16]. Its leaves contain bioactive saponins (bacosides A and B), flavonoids, alkaloids, and other phenolics, which confer their pharmacological effects [17]. Bacopa extracts have been demonstrated to have a wide neuroprotective effect: they protect cholinergic neurons, promote synaptic plasticity (i.e., increasing NMDA receptors and Brain-Derived Neurotrophic Factor (BDNF) expression), scavenge free radicals, prevent Ab fibril formation and toxicity [18,19]. Bacopa also suppresses neuroinflammatory signaling in vitro and in animals. For example, Bacopa therapy suppresses LPS-induced microglial TNF-α and IL-6 release and stimulates antioxidant responses via nuclear factor erythroid 2–related factor 2 (Nrf2) [16]. Such joint effects in inhibition of oxidative stress, suppression of inflammation, and regulation of neurotransmission have been linked with enhanced learning and memory in preclinical models of cognitive impairment [20].

In the current research, we assess the potential of a standardized *B. monnieri* extract (BME) to counteract cognitive impairment and neuroinflammation in a lipopolysaccharide-induced rat model of dementia. This study will further the understanding of the mechanism of action of *B. monnieri* by describing its phytochemicals, antioxidant activity, behavioral, and brain pathology effects. Finally, those results can be used to develop Bacopa-based nutraceuticals or functional foods that promote brain health and reduce cognitive impairment driven by inflammation.

## 2. Results

### 2.1. Bioactive Constituents and Antioxidant Potential of BME

**Figure 1:**
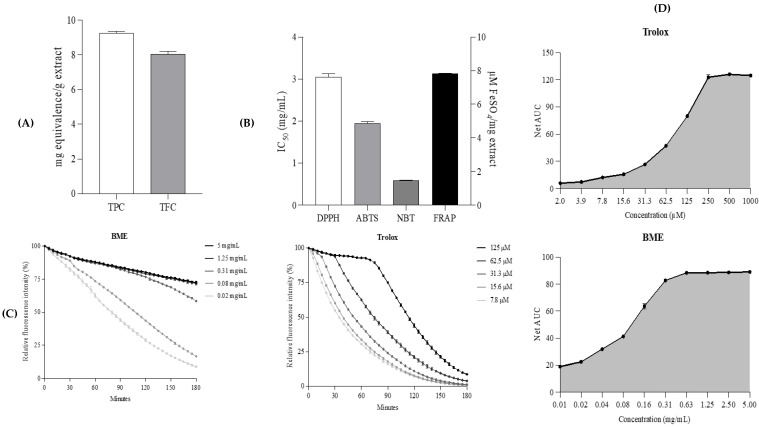
Total phenolic and flavonoid content, along with antioxidant capacities. (**A**) Total phenolic and flavonoid content of BME. (**B**) Antioxidant capacities of BME. (**C**) Kinetic fluorescence decay curves of fluorescein for Trolox and BME. (**D**) Net AUC values for Trolox and BME. Abbreviation: Total phenolic content (TPC) expressed as mg gallic acid equivalence (GAE)/g extract, total flavonoid content (TFC) expressed as mg of catechin equivalence (CAE)/g extract, free radical scavenging activities of DPPH and ABTS, nitroblue tetrazolium (NBT), ferric reducing antioxidant power (FRAP), *Bacopa monnieri* extract (BME).

Total phenolic content (TPC) of BME was 9.27 ± 0.08 mg GAE/g extract, and total flavonoid content (TFC) was 8.04 ± 0.15 mg CAE/g extract (Figure 1A). To establish the antioxidant property of the extract, some complementary assays were also used (Figure 1B; Supplementary Table S1). BME had an IC_50_ of 3.05 ± 0.07 mg/mL in the 2,2-diphenyl-1-picrylhydrazyl (DPPH) assay and 1.95 ± 0.04 mg/mL in the 2,2′-azino-bis (3-ethylbenzothiazoline-6-sulfonic acid (ABTS) assay in radical scavenging assays. The ferric reducing antioxidant power (FRAP) assay, which measures the decrease in ferric ions to ferrous ions, showed a reducing power of 7.83 ± 0.01 μM FeSO4/mg of extract. The extract had a superoxide radical scavenging activity as observed in the nitroblue tetrazolium (NBT) assay with an IC_50_ value of 0.59 ± 0.01 mg/mL. In addition, the oxygen radical antioxidant capacity (ORAC) assay revealed free radical scavenging capacity of 1.17 ± 0.01 µM TE/mg extract. Figure 1C,D also shows the kinetic fluorescence decay curves of fluorescein and the net area under the curve (AUC), which also gives additional evidence on the antioxidant potential of the extract.

### 2.2. Phytochemical Constituents of Bacopa monnieri

**Figure 2:**
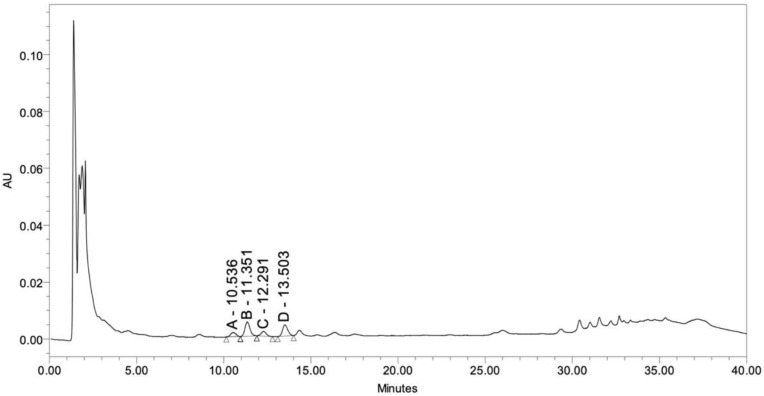
The chromatogram of the high-performance liquid chromatography (HPLC) of bacoside A in BME. The compounds present in the bacoside A mixture, as indicated in the chromatogram, were (A) Bacoside A3, (B) Bacopaside II, (C) Bacopaside X, and (D) Bacopasaponin C.

The active compounds of the BME were determined as a bacoside A mixture and were identified by high-performance liquid chromatography (HPLC), and the subsequent chromatogram is presented in Figure 2. The results were analyzed by giving details of retention times, peak areas, and concentrations of the respective compounds (Supplementary Table S2). The most abundant bacoside component was bacopasaponin C (52.71 ± 0.06 µg/mg), and the subsequent ones were bacopaside II (43.05 ± 0.28 µg/mg), bacoside A3 (33.93 ± 0.17 µg/mg, and bacopaside X (17.44 ± 0.33 µg/mg). These findings indicate the predominance of bacopasaponin C in the bacoside A profile of the extract. Additionally, quantification was performed using peak area integration and external calibration curves; therefore, visual peak height does not directly reflect compound concentration.

### 2.3. Pharyngeal Pumping and Food Clearance Assays in C. elegans

**Figure 3:**
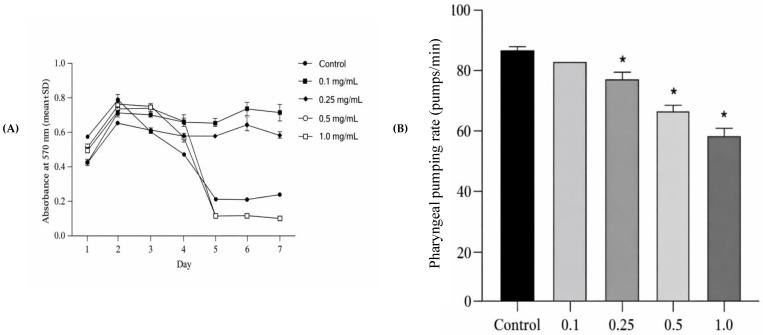
Effects of *Bacopa monnieri* extract on food-intake behavior of *C. elegans* for toxicity evaluation. (**A**) Bacteria clearance assay. (**B**) Pharyngeal pumping rate. The statistical differences with the control group * *p* < 0.05. Extracts of BM at 0.5 and 1.0 mg/mL had a significant effect on the feeding behavior when compared to the control group (*p* < 0.05; Figure 3B). Precisely, the absorbance values showed a decrease on day 1 and then a sharp rise on days 2–4 above control values, and a subsequent decrease towards significantly lower values on days 5–7 (*p* < 0.05; Figure 3B). These alterations in time show that there is a dose-dependent impairment of feeding behavior and patterns of bacterial consumption in *C. elegans*.

*Caenorhabditis elegans* (*C. elegans*) feeding behavior was assessed by determining the rate of pharyngeal pumping in units of contractions of pharyngeal muscles per minute at various concentrations of BME. BME at 0.1 mg/mL had no significant effect on the pumping rate, whereby worms had a rate of 84.00 ± 0.00 pumps/min in comparison to 88.00 ± 2.00 pumps/min in the control group (*p* < 0.05; Figure 3A; Supplementary Table S3a,b). Nevertheless, the greater concentrations (0.25, 0.5, and 1.0 mg/mL) produced a concentration-dependent decrease in the pumping rates (*p* < 0.05; Figure 3A), and the rates decreased to 77.33 ± 4.16, 68.67 ± 1.15, and 57.33 ± 5.03. Toxicity was also evaluated by the food clearance test that involved the quantification of the change in turbidity of *Escherichia coli* (*E. coli*) OP50 in liquid medium over 7 days.

### 2.4. Cognitive Function Improvement of Bacopa monnieri Extract

#### 2.4.1. Effects of *Bacopa monnieri* Extract on Memory Acquisition in LPS-Induced Rats

**Figure 4:**
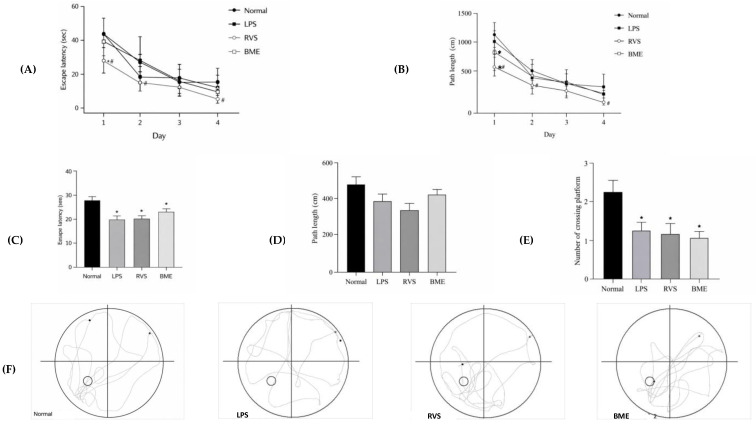
Effects of BM extract on memory and spatial learning in the Morris water maze test of LPS-induced rats. (**A**) Escape latency to find the platform across all training days. (**B**) Path length to find the platform across all training days. (**C**) Escape latency to find the platform on day 5. (**D**) Path length to find the platform on day 5. (**E**) Number of platform crossings on day 5. (**F**) Trajectory of the tracking system in the target quadrant on day 5. The circle indicates the previous location of the hidden platform (that is, the release point of the rat), the line represents the swimming trajectory of the rat, and the dots indicate the starting and ending points of the movement. The statistical differences compared with the normal group * *p* < 0.05; compared with the LPS group # *p* < 0.05. Abbreviations: BME—*Bacopa monnieri* extract, LPS—lipopolysaccharide, RVS—rivastigmine.

The Morris water maze (MWM) was used to assess spatial learning and memory. Across the training period (days 1–4), all groups exhibited progressive reductions in both escape latency and path length to locate the hidden platform (Figure 4; Supplementary Table S4), indicating learning acquisition over time. Although the BME-treated group showed a gradual decline in escape latency from day 1 to day 4 (39.87 ± 3.99, 28.66 ± 4.49, 17.94 ± 3.60, and 10.93 ± 1.38 s), these changes were not significantly different from those observed in the model group (43.14 ± 3.34, 27.40 ± 2.05, 16.58 ± 2.85, and 17.35 ± 2.94 s; *p* > 0.05; Figure 4A). A similar trend was observed for path length, where the BME group demonstrated a non-significant reduction across the training days (909.53 ± 100.74, 537.63 ± 63.95, 448.54 ± 72.91, and 277.57 ± 39.35 cm) compared with the model group (1075.05 ± 74.70, 615.14 ± 62.94, 422.08 ± 60.21, and 400.21 ± 62.42 cm; *p* > 0.05; Figure 4B). The Morris water maze (MWM) was used to assess spatial learning and memory. Training-phase data (days 1–4) were analyzed by two-way mixed (split-plot) ANOVA with group as the between-subjects factor and day as the within-subjects factor. There was a highly significant main effect of training day on both escape latency (F(3, 72) = 58.61, *p* < 0.001, η^2^p = 0.71; Greenhouse–Geisser corrected) and path length (F(3, 72) = 85.92, *p* < 0.001, η^2^p = 0.78; Figure 4A,B), indicating that all groups, including the LPS-induced model group, acquired the task across the training period. The group main effect was also significant for both outcomes (latency: F(3, 24) = 5.81, *p* = 0.004, η^2^p = 0.42; path length: F(3, 24) = 9.32, *p* < 0.001, η^2^p = 0.54), but the group × day interaction did not reach significance (latency: F(9, 72) = 1.50, *p* = 0.166; path length: F(9, 72) = 1.82, *p* = 0.079), indicating that the rate of learning did not differ statistically between groups. The BME-treated group showed a progressive decline in mean escape latency across training days (day 1: 39.87 ± 11.97 s; day 2: 28.66 ± 13.49 s; day 3: 17.94 ± 9.53 s; day 4: 10.93 ± 3.66 s), comparable in trajectory to the LPS-only group (day 1: 43.14 ± 10.58 s; day 2: 27.40 ± 5.43 s; day 3: 16.58 ± 8.06 s; day 4: 17.35 ± 8.31 s); however, post hoc Tukey HSD comparisons revealed no statistically significant difference between BME and LPS on any of the four training days (all *p* > 0.30; Hedges’ g range –0.19 to +0.78; Supplementary Table S6). The same pattern held for path length, with non-significant LPS-vs-BME contrasts across all four days (all *p* > 0.30; Figure 4B). RVS, the positive control, differed significantly from the LPS group on path length on day 1 (mean difference 403 cm, *p* = 0.021, g = 1.94) and day 4 (mean difference 251 cm, *p* = 0.004, g = 2.19), and showed near-significant separation from the LPS group on day 4 escape latency (*p* = 0.057). Descriptive statistics by group and day are provided in the Supplementary Table S6.

In the testing trial, the BME group showed a 23.30% increase in escape latency in the target quadrant compared to the LPS group (22.86 ± 1.32 s vs. 18.54 ± 1.77 s), but the difference was not statistically significant (*p* > 0.05; Figure 4C). Similarly, the path length in the target quadrant was slightly longer in the BME group (423.37 ± 22.93 cm) than in the LPS group (391.24 ± 36.39 cm), a difference of 8.21 percent that was not significant (*p* > 0.05; Figure 4D). Conversely, the number of platform crossings declined by 15.97% in the BME group (1.00 ± 0.15 times) compared with the LPS group (1.19 ± 0.23 times), but this was also not significant (*p* > 0.05; Figure 4E).

These behavioral patterns are further illustrated by representative swim trajectories from the tracking system (Figure 4F). In the probe (testing) trial conducted on day 5, one-way ANOVA revealed that the LPS challenge produced a robust impairment in platform crossings (F(3, 35) = 6.13, *p* = 0.002, η^2^p = 0.34): the normal group performed significantly more platform crossings than the LPS group (mean difference 0.98, *p* = 0.014, Hedges’ g = 1.23), confirming that the LPS-induced cognitive deficit was successfully established. By contrast, no significant differences were observed between the LPS group and either the RVS or BME group on platform crossings (LPS vs. RVS: *p* = 0.999; LPS vs. BME: *p* = 0.919). Mean platform crossings (± SD) were 2.25 ± 0.89 (normal), 1.19 ± 0.65 (LPS), 1.13 ± 0.79 (RVS), and 1.00 ± 0.41 (BME) (Figure 4E). For the remaining probe-trial measures, the omnibus ANOVA did not reach significance for distance in the target quadrant (F (3,36) = 2.14, *p* = 0.112) and was at the threshold for time in the target quadrant (F (3,35) = 2.75, *p* = 0.057). Mean time in the target quadrant was numerically higher in the BME group than in the LPS group (22.86 ± 3.96 s vs. 18.54 ± 5.01 s, Hedges’ g = –0.59), and mean distance in the target quadrant was likewise numerically higher in the BME group (423.37 ± 68.80 cm vs. 391.24 ± 96.28 cm, g = –0.25), but neither contrast reached statistical significance in post hoc comparisons (*p* = 0.565 and *p* = 0.944, respectively; Figure 4C,D). Representative swim trajectories of the tracking system are shown in Figure 4F. Full statistical output, including F-statistics, exact *p*-values, and effect sizes for all pairwise contrasts, is provided in Supplementary Table S6.

#### 2.4.2. Effects of *Bacopa monnieri* Extract on Memory Retention in LPS-Induced Rats

**Figure 5:**
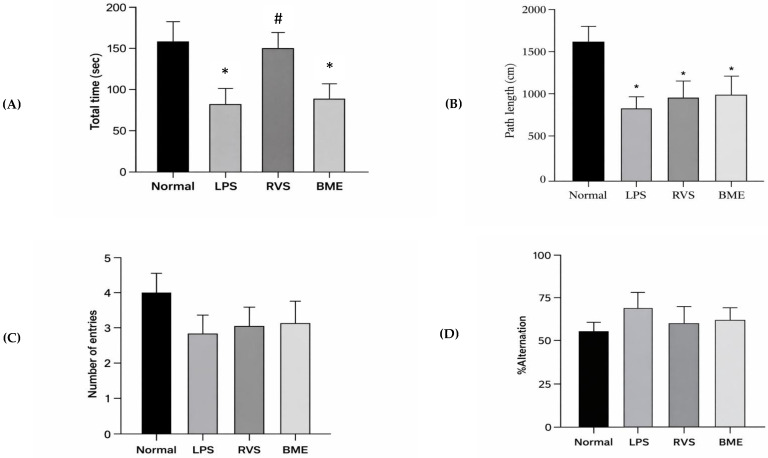
Effects of *Bacopa monnieri* extract on working memory and short-term memory in the Y-maze test of LPS-induced rats. (**A**) Total time spent in the novel arm. (**B**) Path length in the novel arm. (**C**) Number of entries into the novel arm. (**D**) Spontaneous alternation behavior. The statistical differences compared with the normal group * *p* < 0.05; compared with the LPS group # *p* < 0.05. Abbreviations: BME—*Bacopa monnieri* extract, LPS—lipopolysaccharide, RVS—rivastigmine.

The Y-maze test was conducted to evaluate working and short-term memory performance. Compared with the LPS, the BME-treated group exhibited modest, non-significant increases in exploratory behavior, including total time spent in the maze (77.40 ± 18.75 vs. 72.29 ± 15.63 s), path length (1011.61 ± 206.49 vs. 829.42 ± 112.22 cm), and number of entries into the novel arm (3.14 ± 0.74 vs. 2.43 ± 0.53 times), corresponding to increases of 7.07%, 21.96%, and 29.22%, respectively (*p* > 0.05; Figure 5A–C; Supplementary Table S5). In contrast, spontaneous alternation behavior, an indicator of working memory, was slightly reduced in the BME group (60.38 ± 6.05%) compared with the LPS (69.46 ± 8.53%), representing a 0.87% decrease, although this difference was not statistically significant (*p* > 0.05; Figure 5D). Overall, these findings suggest that while BME treatment may enhance exploratory activity, it did not produce a significant improvement in working memory under the conditions tested.

### 2.5. Effects of BME on LPS-Induced Neuroinflammation in Rat Brain

**Figure 6:**
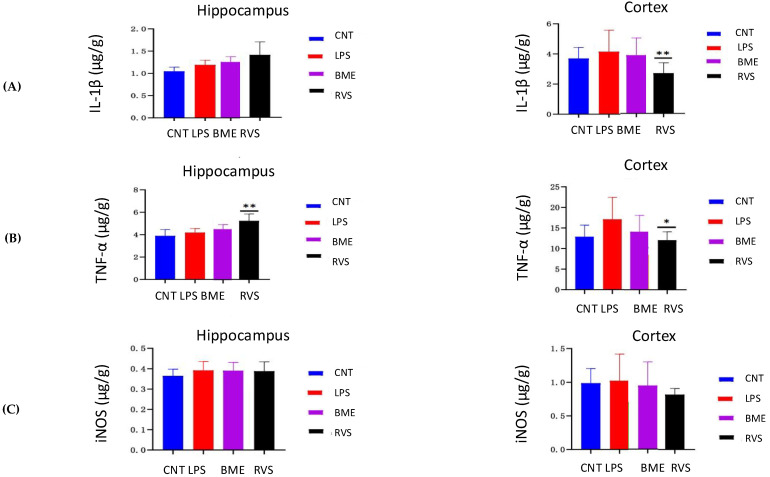
Effects of BME treatment on pro-inflammatory markers in rat hippocampus and cortex. Levels of interleukin-1 beta (IL-1β), tumor necrosis factor-alpha (TNF-α), and inducible nitric oxide synthase (iNOS) were quantified in the hippocampus and cortex of rats across experimental groups. (**A**) IL-1β concentrations, (**B**) TNF-α concentrations, and (**C**) iNOS levels are presented for control (CNT), lipopolysaccharide-treated (LPS), *Bacopa monnieri* extract-treated (BME), and rivastigmine (RVS) groups. Data are expressed as mean ± standard deviation (SD) for n = 10 rats per group. Statistical analysis was performed using one-way analysis of variance (ANOVA). Asterisks indicate statistically significant differences compared with the LPS group (* *p* < 0.05, ** *p* < 0.01). Error bars represent standard deviation.

As shown in Figure 6, LPS administration decreased IL-1β levels in the hippocampus by 13.74%, while increasing cortical IL-1β levels by 12.15%. Treatment with BME and RVS elevated hippocampal IL-1β levels by 5.50% and 18.03%, respectively. In contrast, both treatments reduced cortical IL-1β levels by 6.65% and 35.45% (*p* < 0.01). LPS increased TNF-α levels in the hippocampus and cortex by 7.53% and 32.47%, respectively. Compared with the LPS group, BME and RVS further increased hippocampal TNF-α levels by 7.52% and 24.93% (*p* < 0.01). However, both treatments significantly reduced TNF-α levels in the cortex by 17.37% and 29.60% (*p* < 0.05). iNOS levels were elevated in the hippocampus and cortex of the LPS group by 7.47% and 3.66%, respectively, compared with the control group. Relative to the LPS group, BME slightly reduced iNOS levels in the hippocampus (0.33%) and cortex (7.24%), while RVS decreased hippocampal and cortical iNOS levels by 0.91% and 20.15%, respectively.

COX-2 expression increased in the hippocampus and cortex of the LPS group by 14.94% and 38.29%, respectively, compared with the control group. BME reduced hippocampal COX-2 levels by 4.24%, whereas RVS slightly increased them by 2.20%. In the cortex, BME and RVS decreased COX-2 levels by 1.08% and 19.27%, respectively; however, these changes were not statistically significant. AChE expression in the hippocampus and cortex decreased by 21.64% and 3.16%, respectively, in the LPS group compared with the control group.

**Figure 7:**
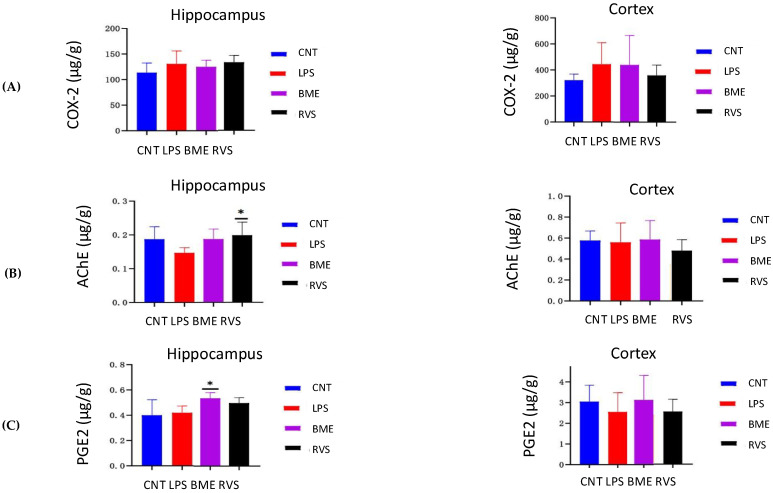
Effects of BME treatment on COX-2, AChE, and PGE_2_ levels in rat hippocampus and cortex. Cyclooxygenase-2 (COX-2), acetylcholinesterase (AChE), and prostaglandin E_2_ (PGE_2_) levels were measured in the hippocampus and cortex of rats across experimental groups. (**A**) COX-2 concentrations, (**B**) AChE activity, and (**C**) PGE_2_ levels are shown for control (CNT), lipopolysaccharide-treated (LPS), *Bacopa monnieri* extract-treated (BME), and rivastigmine (RVS) groups. Data are expressed as mean ± standard deviation (SD) for n = 10 rats per group. Statistical analysis was performed using one-way analysis of variance (ANOVA). Asterisks indicate statistically significant differences compared with the LPS group (* *p* < 0.05). Error bars represent standard deviation.

Treatment with BME and RVS significantly increased hippocampal AChE levels by 27.74% and 27.43% (*p* < 0.05). Conversely, cortical AChE levels were reduced by 4.86% (*p* < 0.05) and 14.04%, respectively. PGE2 levels increased in the hippocampus by 4.95% but decreased in the cortex by 16.42% in the LPS group relative to controls. Compared with the LPS group, BME significantly increased PGE2 levels in both the hippocampus (28.11%, *p* < 0.05) and the cortex (22.84%). RVS increased hippocampal PGE2 levels by 18.55% but had a minimal effect on cortical PGE2 levels (0.17%). Overall, LPS exerted relatively modest effects on IL-1β, TNF-α, iNOS, and COX-2 levels, and BME showed limited modulation of these markers in the hippocampus. In contrast, more pronounced effects were observed for AChE and PGE2, suggesting a differential sensitivity of cholinergic and prostaglandin pathways under these experimental conditions (Figure 7).

## 3. Discussion

This study examined the impact of BM extract, a medicinal plant known to have neuroprotective and antioxidant activity, on cognitive performance in a model of LPS-induced dementia. The results of the study indicate that BM extract can be useful in the preservation of cognitive abilities and neuroinflammation, which means that it can be used as a preventive or as an adjunctive treatment of dementia. Phytochemical studies revealed that ethanolic BM extract contained phenolic and flavonoid compounds, which are significant sources of antioxidant activity. As anticipated, the total phenolic content was more than the total flavonoid content since flavonoids form a subgroup in the general phenolic group [21,22]. The observation in phytochemical profiles made in this study, as opposed to the previous reports, could be due to variations in the plant part, harvesting conditions, and the solvent of choice. Polar solvents (acetone or methanol) tend to extract phenolic compounds more efficiently than ethanol, which was used in the current study [23,24,25]. Since phenolics and flavonoids can neutralize free radicals, give electrons or hydrogen atoms, and chelate metals, their appearance has a great biochemical explanation of the antioxidant effects shown in this study [26].

BM extract has an antioxidant profile that was verified using a variety of in vitro assays. The DPPH and ABTS outcomes indicated that the extract can counteract free radicals via hydrogen atom transfer reaction, as well as single electron transfer reaction, in agreement with phenolic antioxidant behavior [27,28,29]. The reduced IC_50_ in ABTS compared to DPPH could be due to the fact that ABTS has a higher solvent range and a higher rate of reaction. This activity was further supported by further mechanistic activity: FRAP revealed electron-donating ability by reduction in ferric ions, NBT revealed scavenging of superoxide, and OARC revealed scavenging of peroxyl radical by hydrogen donation [30,31,32,33]. These results indicate that BM extract works primarily by radical scavenging and reducing actions, thus preventing the extent of oxidative destruction of lipids, proteins, deoxyribonucleic acid (DNA), and other cellular molecules. Conversely, no metal chelating activity was observed, which may have been due to the possibility of the presence of hydroxyl groups in the phenolic compounds in other interactions, or that the hydroxyl groups of the ferrozine assay were not available to bind with ferrous ion [34,35,36].

Bacoside A has also been identified as the major bioactive substance by phytochemical profiling, along with bacoside A3, bacopaside II, bacopaside X, and bacopasaponin C [37]. The lower bacoside content compared with some earlier studies is likely explained by variations in solvent polarity, plant part used, and growing conditions. Methanol is generally more polar than ethanol and thus tends to extract bacosides more effectively, whereas leaves and stolons tend to be the most abundant plant parts [38,39,40,41,42]. These variations emphasize the importance of extraction conditions when comparing BM preparations across studies.

The dose–response effect of screening for safety in *C. elegans* was dose-dependent. BM extract did not affect pharyngeal pumping at lower concentrations, implying no acute toxicity at that level. Nevertheless, higher concentrations reduced pharyngeal contractions and feeding behavior, indicating potential toxicity at high doses [43,44,45]. Food clearance tests also showed an initial decrease in feeding at high concentrations, followed by recovery over time, suggesting a transient effect rather than toxicity [46,47,48,49,50,51,52]. Collectively, these assays suggest that BM extract is well tolerated at lower doses yet may have concentration-dependent effects on feeding and neuromuscular activity.

In the present study, the LPS challenge produced the expected cognitive deficit, validating the model: in post hoc comparisons, the normal group differed significantly from the LPS group on MWM platform crossings (Hedges’ g = 1.23, *p* = 0.014), Y-maze distance in the novel arm (g = 1.57, *p* = 0.009), and Y-maze time in the novel arm (g = 1.48, *p* = 0.012). LPS is widely used to induce systemic and central inflammation by activating toll-like receptor 4 (TLR4), leading to microglial activation, increased pro-inflammatory cytokine release, oxidative stress, impaired synaptic function, and memory loss, symptoms similar to those in neurodegenerative diseases [53,54]. Pre-treatment with BME at 70 mg/kg did not produce a statistically significant rescue of any behavioral endpoint relative to the LPS-only group. Across the key LPS-vs-BME pairwise contrasts in the MWM (training-phase escape latency and path length on each of days 1–4, plus the three probe-trial measures) and the Y-maze (distance, time, and entries), no comparison reached *p* < 0.05; the largest effect size in the protective direction was for day 4 path length (Hedges’ g = 0.80, *p* = 0.306). Several other contrasts showed small-to-medium effect sizes in the expected protective direction (e.g., probe-trial time in target quadrant, g = −0.59; Y-maze distance, g = −0.43). Taken together, the in vivo behavioral results indicate a directional trend consistent with a protective effect but do not provide statistical evidence that BME rescued LPS-induced cognitive impairment under the conditions tested. The positive control, rivastigmine, showed isolated significant effects on path length on days 1 and 4 but did not consistently outperform the LPS group on the probe-trial or Y-maze measures, which is consistent with the limited efficacy commonly reported for cholinesterase inhibitors in acute LPS models.

The neural substrates likely relevant to the present behavioral measures are well established. Performance in the MWM is supported primarily by hippocampal circuitry, particularly Cornu Ammonis-1 (CA1) and Cornu Ammonis-3 (CA3), which play a central role in spatial learning and memory [55,56]. In our hands, the LPS-only group showed clear deficits in platform crossings and in novel-arm distance and time, consistent with neuroinflammation disrupting these circuits. Pre-treatment with BME produced effect sizes in the protective direction for several measures (probe-trial time in the target quadrant, Hedges’ g = −0.59; day 4 path length, g = 0.80) but did not produce statistically significant rescue at the tested dose. Previous reports of stronger BME-mediated cognitive benefit in hypoxia-induced and chemically induced memory-impairment models [57,58] used different injury paradigms, treatment durations, and dose ranges. By contrast, novel-arm exploration and spontaneous alternation in the Y-maze depend on hippocampal–prefrontal networks that govern attention and working memory [59,60,61,62]. The discrepancy between those reports and our findings underscores the importance of injury model, dose, and treatment timing in determining detectable efficacy. The directional but non-significant trends observed here are broadly consistent with earlier literature suggesting that BME may act more effectively as a preventive than as a reversal agent in cognitive-impairment models [63,64,65], and would warrant follow-up in larger, dose-ranging studies before firmer mechanistic conclusions are drawn.

Furthermore, our findings reveal a region-specific and pathway-selective modulation of neuroinflammatory and cholinergic markers following LPS challenge and subsequent treatment with BME. Such differential responses between the hippocampus and cortex are increasingly recognized in experimental neuroinflammation models and may reflect intrinsic heterogeneity in microglial priming, neuronal vulnerability, and blood- brain barrier dynamics across brain regions. The relatively modest and regionally divergent changes observed in IL-1β in the present study, characterized by a decrease in the hippocampus and an increase in the cortex, suggest a temporally dynamic or compensatory regulatory mechanism. Recent studies indicate that hippocampal cytokine responses to systemic LPS may exhibit biphasic kinetics, with early suppression or exhaustion phases following acute activation, particularly under conditions of sustained neuroimmune stimulation. Conversely, cortical regions may sustain prolonged cytokine elevation due to differential microglial activation thresholds and cytokine clearance rates [66].

The observed increase in TNF-α in both regions following LPS administration aligns with established literature demonstrating its central role in mediating neuroinflammation and synaptic dysfunction [66]. Notably, the further elevation of hippocampal TNF-α following BME treatment, despite concomitant reductions in the cortex, may reflect a context-dependent immunomodulatory effect rather than a purely anti-inflammatory action. Emerging evidence suggests that moderate TNF-α signaling in the hippocampus can support synaptic plasticity and neuroprotection under certain conditions, particularly via TNF receptor 2 (TNFR2)-mediated pathways [67]. Thus, the elevation observed here may not necessarily indicate detrimental inflammation but rather a recalibration of neuroimmune signaling. The modest elevation of iNOS following LPS exposure and its slight attenuation by BME are consistent with previous reports highlighting nitric oxide (NO)-mediated oxidative stress as a secondary component of LPS-induced neurotoxicity and BME as an ameliorative agent [18]. Similarly, the increase in COX-2 expression following LPS administration corroborates its role as an inducible enzyme in neuroinflammation, contributing to prostaglandin synthesis and inflammatory amplification [67]. The relatively weak modulation of COX-2 by BME suggests that this pathway may be less sensitive under the present experimental conditions or that it requires longer intervention durations for significant suppression. This observation is supported by reports indicating that COX-2 expression is tightly regulated and may not always correlate directly with upstream cytokine changes [68].

In contrast to the modest cytokine and enzyme responses, the pronounced alterations in AChE and PGE2 highlight a stronger involvement of cholinergic and prostaglandin pathways. The reduction in AChE following LPS exposure is consistent with a compensatory increase in acetylcholine availability aimed at counteracting inflammation via the cholinergic anti-inflammatory pathway. The restoration and significant elevation of hippocampal AChE levels by BME may reflect normalization of cholinergic tone following attenuation of inflammatory stress [69]. The marked increase in PGE2 levels following BME treatment in both brain regions is particularly noteworthy. While PGE2 is traditionally considered pro-inflammatory, recent studies have demonstrated its dual role, including neuroprotective and inflammation-resolving functions depending on receptor subtype engagement (Prostaglandin E2 receptor subtype 2 or 4- EP2 vs. EP4) and local concentration gradients [70]. The elevation of PGE2 observed here may therefore represent an adaptive, pro-resolving response rather than exacerbation of inflammation. This is further supported by the limited changes in COX-2, suggesting selective downstream modulation rather than global pathway activation.

Taken together, the most robust positive outcomes of the present study are the phytochemical characterization and the in vitro antioxidant profile of this standardized BM extract, which clearly demonstrates radical scavenging and reducing capacity attributable to its phenolic, flavonoid, and bacoside constituents. The in vivo behavioral data, while showing several non-significant trends in the protective direction, do not provide statistical evidence that BME at 70 mg/kg rescues LPS-induced cognitive impairment in this model. Additionally, BME demonstrated limited effects on classical inflammatory markers but exerted more pronounced modulation of prostaglandin signaling.

Hypothetically, the antioxidant and anti-inflammatory properties observed in vitro could underlie a protective effect in vivo at higher doses, longer treatment durations, or in chronic-injury paradigms; this hypothesis remains to be tested. Future research is needed to characterize the active ingredients in greater detail, to conduct formal in vivo dose–response evaluation, and to test whether formulation strategies such as microencapsulation can improve in vivo bioavailability and efficacy relative to the crude extract.

Several limitations of the present study should be acknowledged in interpreting the in vivo findings. First, the study used a single dose of BME (70 mg/kg, p.o.); a formal dose–response evaluation was beyond the scope of this work, and the absence of a higher-dose arm may have limited the ability to detect a treatment effect. Second, per-group sample sizes for the behavioral endpoints (n = 6–11 per outcome after exclusions) provided limited statistical power to detect medium-sized effects, and several LPS-vs-BME contrasts showed effect sizes in the small-to-medium range (Hedges’ g up to 0.80) without reaching *p* < 0.05; this is best interpreted as a power-limited finding rather than as definitive evidence of no effect. Third, the LPS challenge used (0.175 mg/kg, daily i.p. for 7 days following a 14-day pre-treatment window) is a relatively acute systemic inflammation paradigm and may not fully recapitulate the chronic, low-grade neuroinflammation that characterizes age-related cognitive decline. Longer pre-treatment, chronic LPS exposure, or alternative neuroinflammatory or neurodegenerative models may be more sensitive to BME’s mechanism of action. Fourth, corroboration of the proposed mechanism through molecular and tissue-level endpoints is identified as a priority for follow-up work.

## 4. Materials and Methods

### 4.1. Plant Materials

The standardized alcoholic extract of *Bacopa monnieri* with 21.24% bacosides (BCP-2209147) was bought from AP Operations Co., Ltd., Sri Racha, Thailand. The extract was pale brown to greenish brown. No further extraction or processing of the plant material was performed in our laboratory. According to the supplier’s Certificate of Analysis (CoA, lot BCP-2209147), the extract is prepared from the aerial parts of *Bacopa monnieri* using a hydroethanolic solvent system and standardized to a total bacoside content of 21.24% (*w*/*w*) of the dried plant material. Upon receipt, the extract was stored in tightly sealed amber containers at 4 °C, protected from light and moisture, and used within the manufacturer’s specified shelf life. A single batch was used for all experiments reported in this study to eliminate batch-to-batch variability. The use of a pre-standardized, commercially sourced extract was a deliberate methodological choice to ensure consistent bacoside content across experiments and to permit comparison with previous studies that have employed similarly standardized BME preparations.

### 4.2. Determination of Total Phenolic and Total Flavonoid Contents

#### 4.2.1. Total Phenolic Content

The TPC of the extract was measured as described previously [71] with modifications. Briefly, 20 µL of the 5 mg/mL sample was added to a 96-well plate, followed by 80 µL of distilled water and 6 µL of 5% sodium nitrite (NaNO_2_) (*w*/*v*), and incubated in the dark for 6 min at room temperature. Then add 6 µL of 10% aluminum chloride (AlCl_3_) (*w*/*v*) and incubate the mixtures in the dark for another 6 min. Finally, 40 µL of 1 M sodium hydroxide (NaOH) was added, and the absorbance was read at 510 nm. The TFC is expressed in milligrams of catechin equivalents per gram of extract (mg CAE/g extract).

#### 4.2.2. Total Flavonoid Content

The TFC of the extract was determined using the aluminum chloride method with some modifications [71]. Briefly, 20 µL of the 5 mg/mL sample was added to a 96-well plate, followed by 80 µL of distilled water and 6 µL of 5% NaNO_2_ (*w*/*v*), and incubated in the dark for 6 min at room temperature. Then add 6 µL of 10% AlCl_3_ (*w*/*v*) and incubate the mixtures in the dark for another 6 min. Finally, 40 µL of 1 M NaOH was added, and the absorbance was measured at 510 nm. The TFC was expressed as milligrams of catechin equivalents per gram of extract (mg CAE/g extract).

### 4.3. Determination of Antioxidant Capacities

#### 4.3.1. Metal Chelating Assay

The MCA was used to assess the presence of excess transition metals, utilizing ethylenediaminetetraacetic acid (EDTA) as the standard and some method modification [72]. To a 96-well plate, 40 µL of the 5 mg/mL sample was added with 80 µL of 0.25 mM ferrous sulfate (FeSO_4_), and these samples were left in the dark for 10 min at room temperature. A volume of 80 µL of 0.5 mM ferrozine was then added, and the mixture was incubated for 10 min at room temperature. The absorbance was read at 562 nm. The metal chelating activity was calculated as follows:

MCA %inhibition = [(OD of sample − OD of blank)/OD of control] × 100.

#### 4.3.2. DPPH Free Radical Scavenging Assay

Colorimetric DPPH assay, with Trolox as standard, and some modifications of the method were used [72]. To a 96-well plate, 20 µL of the 5 mg/mL sample was added, and then 180 µL of the 80 µM DPPH ethanol solution was added. The samples were mixed and allowed to stand in the dark at room temperature for 5 min. The mixtures were left to incubate for 5 min, and then absorbances were read at 490 nm. DPPH free radical scavenging activity was measured using the following formula:

DPPH %inhibition = [(OD of sample − OD of blank)/OD of control] × 100.

#### 4.3.3. ABTS Free Radical Scavenging Assay

The ABTS test, a colorimetric assay with Trolox as the standard, with slight modifications [72], was used for the ABTS free radical scavenging assay. ABTS solution was prepared by adding 1:1 (*v*/*v*) of 2 mM ABTS and 2.45 mM potassium persulfate (K_2_S_2_O_8_) solution and left in the dark at room temperature for 16 h. Then the ABTS solution was diluted with 50 mM phosphate buffer (pH 7.4) to achieve an absorbance of 0.70 ± 0.02 at 734 nm. Into the 96-well plate was added 20 µL of the 5 mg/mL sample solution and 200 µL of ABTS solution. Then the solutions were mixed well and allowed to stand in the dark at room temperature for 6 min. The absorbance was then measured at 743 nm. The ABTS free radical scavenging activity was determined by the following equation:

ABTS % inhibition = [(OD of sample − OD of blank)/OD of control] × 100.

#### 4.3.4. Ferric Reducing Antioxidant Power Assay

The FRAP assay was used to measure the reduction in ferric ions to ferrous ions, using Ferrous sulfate heptahydrate (FeSO_4_·7H_2_O) as the standard and some slight modifications [71,72]. The FRAP solution was a 10:1:1 mixture of 300 mM acetate buffer, 10 mM 2,4,6-Tripyridyl-s-triazine (TPTZ), and 20 mM ferric chloride (FeCl_3_). A 96-well plate was filled with 20 µL of the 5 mg/mL sample and 180 µL of FRAP solution. This mixture was mixed thoroughly and incubated in the dark for 30 min. After the incubation, the absorbance was measured at 593 nm. The FRAP was calculated as micromoles of FeSO_4_ per milligram of sample (µM FeSO_4_/mg extract).

#### 4.3.5. Nitroblue Tetrazolium Assay

Scavenging activity of superoxide radicals was determined using the NBT assay, with the standard as Trolox and some modification of the method [72]. The solution was prepared by combining 1 mL of 1 mg/mL NBT, 1 mL of 10 mg/mL riboflavin, 1 mL of 10 mM methionine, and 2 mL of 150 mM phosphate buffer (pH 7.4). In a 96-well plate, 100 µL of a 5 mg/mL sample was mixed with 100 µL of NBT solution. The mixtures were thoroughly mixed and incubated under illumination with fluorescent lamps (20 W) at room temperature for 10 min. The absorbance was measured at 560 nm after the incubation. The superoxide radical scavenging ability was determined by the equation:

NBT %inhibition = [(OD of sample − OD of blank)/OD of control] × 100.

#### 4.3.6. Oxygen Radical Antioxidant Capacity

The antioxidant scavenging of free radicals using the hydrogen atom transfer mechanism was measured by the ORAC assay (using Trolox as the standard) [72]. The fluorescein solution was prepared by diluting 40 nM fluorescein in 7.5 mM phosphate buffer, pH 7.4 (1:9 *v*/*v*). In each well of the 96-well plate, 25 µL of the 5 mg/mL sample extract was mixed with 150 µL of fluorescein solution and 25 µL of 153 mM 2,2′-azobis(2-amidinopropane) dihydrochloride (AAPH). The mixture was well mixed and incubated in the dark at 37 °C for 15 min. After incubation, the absorbance (emission—523 nm, excitation—485 nm) was measured every 5 min for 3 h. The scavenging of free radicals was calculated as micromoles of Trolox equivalents per milligram of extract (mg TE/mg extract).

### 4.4. Analytical Equipment for 96-Well Plate Assays

The TPC, TFC, and antioxidant activities were measured using a TECAN Spark microplate reader (TECAN Group Ltd., Männedorf, Switzerland) with absorbance through colorimetric and fluorescent intensity.

### 4.5. Evaluation of Toxicity

#### 4.5.1. Food Clearance Assay

The 10 µL of L1 stage *C. elegans* from the synchronized S-medium buffer was mixed into a 96-well plate together with 80 µL of *E. coli* OP50 (OD570 nm = 0.6) and 10 µL of extracts at 0.1, 0.25, 0. Then incubated at 20 °C, and the absorbance was read at 570 nm for 7 days [72].

#### 4.5.2. Pharyngeal Pumping Assay

The 10 µL of *C. elegans* L4 stage from the synchronized S-medium buffer was added to a 96-well plate with 80 µL of *E. coli* OP50 (OD570 nm = 0.6) and 10 µL of the extracts at concentrations of 0.1, 0.25, 0.5, and 1.0 mg/mL. Thus, after incubation at 20 °C for 24 h, *C. elegans* were counted at random by the contraction and relaxation of the pharyngeal muscles under a microscope for 1 min and repeated three times. The data were expressed as the number of *C. elegans* feeding per minute [72].

### 4.6. Determination of Phytochemical Compounds

The HPLC analysis was carried out using a Waters/Acquity Arc system with a photodiode array detector (PDA) (HPLC 2998, 800 nm) from Waters Corporation, Milford, MA, USA. The Kinetex EVO C18 reverse-phase column (150 mm × 2.1 mm, 5 μm) from Phenomenex, Torrance, CA, USA, was used for separation in binary gradient mode (solvent A: acetonitrile and solvent B: orthophosphoric acid). The gradient program was as follows: 0–25 min, 30–40% A; 25–30 min, 40–60% A; 30–32 min, 60% A; 32–35 min, 60–30% A; and 35–40 min, 30% A. The temperature of the column was set at 30 °C. The flow rate was 0.2 mL/min, and the injection volume for both the sample and the standard solutions was 5 µL. Chromatograms were acquired at a detector wavelength of 205 nm [73]. Four bacoside reference standards were used to identify and quantify the major bacoside A constituents in the extract, including bacoside A3 (peak A), bacopaside II (peak B), bacopaside X (peak C), and bacopasaponin C (peak D). All four standards were obtained from Sigma-Aldrich (Merck KGaA, Darmstadt, Germany). Stock solutions were prepared in HPLC-grade methanol and serially diluted in the same solvent to obtain seven calibration concentrations (12.5, 25, 50, 100, 250, 500, and 1000 ppm) for each standard. Calibration curves were constructed by linear regression of integrated peak area against nominal concentration. The regression equations and coefficients of determination (R^2^) for the four standards were: bacoside A3, y = 794.21x + 5729.10, R^2^ = 0.9959; bacopaside II, y = 2133.67x + 7204.86, R^2^ = 0.9975; bacopaside X, y = 1456.31x + 8350.58, R^2^ = 0.9963; and bacopasaponin C, y = 1645.07x − 4583.33, R^2^ = 0.9994. Limits of detection (LOD) and quantification (LOQ) were calculated as 3.3·σ/slope and 10·σ/slope, respectively, where σ is the standard error of the residuals of each calibration curve. Full calibration parameters, including LOD and LOQ values for each compound, the supplier information, and the raw calibration data points, are provided in Supplementary Table S7, and a representative chromatogram of the four standards co-injected at a mid-range concentration is shown in Supplementary Figure S1.

### 4.7. Animals

Male Sprague-Dawley rats (specific pathogen-free (SPF) grade), whose weights are between 280 and 300 g, were purchased from Beijing Huafukang Biotechnology Co., Ltd., Beijing, China. Animal activities followed the guidelines of the Institutional Animal Care and Use Committee (IACUC) of Chengde Medical College, China (Approval No. CDMULAC-20240715-033). The animals were confined to groups of five in a cage and fed 20 g of food per day. The conditions of housing were kept in controlled laboratory conditions, such as 25 °C ambient temperature and a 12 h light–dark cycle. All animal experiments followed the ARRIVE (Animal Research: Reporting of In Vivo Experiments) guidelines.

### 4.8. Animal Experiment

Animals were randomized into 4 groups with 10 rats in each group (n = 40). Oral or intraperitoneal injection of the treatments was given for 14 days, and intraperitoneal injection of LPS was given for 7 days (days 15–21). The animal groups included: (1) normal animals receiving oral administration of normal saline (0.2 mL/kg), (2) animals receiving an injection of LPS (0.175 mg/kg), (3) animals receiving an injection of LPS (0.175 mg/kg) and rivastigmine (RVS; 0.175 mg/kg), and (4) animals receiving an injection of LPS (0.175 mg/kg) and oral administration of BME (70 mg/kg). Behavioral studies were performed with the Morris water maze test (days 22–26) and Y-maze test (day 27). Animals were sacrificed on day 28 under anesthesia, which was induced by intraperitoneal injection of a mixture of ketamine and xylazine to ensure that the animal was properly sedated and to reduce any pain or suffering. When the animal was under deep anesthesia, blood was withdrawn from the heart [74,75]. Rivastigmine (Lot: F1509072) was provided by Shanghai Aladdin Biochemical Technology Co., Ltd., Shanghai, China and LPS (Lot: 254118) was provided by MedChemExpress LLC, Monmouth Junction, NJ, USA.

### 4.9. Behavioral Assessment

#### 4.9.1. Morris Water Maze Test

MWM was carried out with adaptive swimming for 1 day, lasting for 120 s without recording. On days 1 to 4, swimming on the hidden platform was 60 s for 4 sessions (twice in the morning, twice in the afternoon). The escape distance and escape latency from the starting point to the platform were measured, and the means were calculated. On day 5, the platform was removed from the target quadrant, and the total distance, total time, and the total number of times the animal entered the target quadrant were recorded [76,77].

#### 4.9.2. Y-Maze Test

For the Y-maze test, the maze consists of the starting arm (A), the other arm (B), and the novel arm (C). Place a door in the novel arm (C) to prevent the rats from entering. Keep the rats at the starting point of the starting arm (A) for 10 min. After removing the partition in the novel arm (C), observe the total distance, total time, and frequency of entries that the rats make into the novel arm (C) for 5 min. The level of spontaneous alteration behavior was determined by the following equation [76,77,78]:

%alteration = [(number of alterations)/(total arm entries − 2)] × 100.

### 4.10. Evaluation of the Effects of BME on Neuroinflammatory Markers in LPS-Induced Rats

Cortex and hippocampus tissues (100–150 mg) were collected, minced, and homogenized in phosphate-buffered saline (PBS, 0.01 M, pH 7.4) using a glass homogenizer. Samples were rapidly frozen in liquid nitrogen for storage and subsequently thawed and maintained at 2–8 °C. Homogenization was performed at 2000–3000 rpm for 20 min, followed by centrifugation, and the supernatant was collected for analysis. Levels of acetylcholinesterase (AChE), inducible nitric oxide synthase (iNOS), interleukin-1β (IL-1β), prostaglandin E2 (PGE2), and tumor necrosis factor-α (TNF-α) were quantified using ELISA kits (Genemei Biotech Co., Ltd., Guangzhou, China). For cyclooxygenase-2 (COX-2) measurement, cortex/hippocampus tissue was homogenized with pre-cooled PBS (1:9, *w*/*v*), centrifuged at 5000× *g* for 5 min, and the supernatant was analyzed using ELISA kits (Elabscience Bionovation Inc., Wuhan, China). All assays were conducted according to the manufacturer’s instructions using a microplate reader (440–460 nm).

### 4.11. Data Analysis

All in vitro assays were conducted in triplicate. Descriptive statistics are shown as mean ± standard deviation (SD) throughout, including in the text, figures, and tables. The choice of statistical test was consistent with the experimental design. For the Morris water maze training data (escape latency and path length over days 1–4), which are repeated measures on the same subjects, a two-way mixed ANOVA was used with group as the between-subjects factor (4 levels: normal, model, RVS, BME) and day as the within-subjects factor (4 levels: days 1–4). Mauchly’s test assessed sphericity; the Greenhouse–Geisser correction’s *p*-value is reported if sphericity is violated. For probe-trial measures on day 5 (distance, time in the target quadrant, number of platform crossings) and Y-maze indices (distance, time, entries in the novel arm), which are single measurements, a one-way ANOVA was applied with group as the between-subjects factor. Homogeneity of variance was checked with Levene’s test. Post hoc comparisons were performed using Tukey’s HSD test. Effect sizes are reported as partial eta-squared (η^2^p) for omnibus effects and Hedges’ g for pairwise analyses. Analyses were conducted using Python 3 with pingouin (v0.6.1), SciPy, and verified in SPSS v25 and GraphPad Prism v9. Statistical significance was set at α = 0.05, two-tailed. The full statistical output, including F-statistics, degrees of freedom, *p*-values, effect sizes, and post hoc results, is in Supplementary Table S6.

For ELISAs (IL-1β, TNF-α, iNOS, COX-2, AChE, PGE_2_), each biological sample was assayed in triplicate wells, and the mean of the technical replicates was used as the value for that biological replicate. For the food clearance and pharyngeal pumping assays, each biological replicate represents an independent experimental run, and measurements were averaged. Statistical analyses were performed in SPSS 25 (IBM Corp., Armonk, NY, USA). Normality was assessed with the Shapiro–Wilk test and Q-Q plots, and homogeneity of variances with Levene’s test. One-way ANOVA followed by Tukey’s multiple-comparison test was used when assumptions were met; otherwise, the Kruskal–Wallis test was applied. Post hoc comparisons used Tukey’s HSD when variances were equal and the Games–Howell test when they were not. A *p*-value of less than 0.05 was considered statistically significant.

## 5. Conclusions

The standardized BME used in this study contained appreciable concentrations of key phytochemical constituents, including bacoside A components (bacopasaponin C, bacopaside II, bacoside A3, and bacopaside X), phenolics, and flavonoids, and exhibited robust in vitro antioxidant activity via both single-electron transfer (SET) and hydrogen atom transfer (HAT) mechanisms. The extract was well tolerated in *C. elegans* at lower concentrations, with mild, reversible effects on feeding behavior at higher concentrations. In the LPS-induced rat model of cognitive impairment, the LPS challenge produced the expected behavioral deficits relative to normal controls, validating the model. Pre-treatment with BME (70 mg/kg, p.o.) at the single dose tested did not produce statistically significant rescue of any behavioral endpoint relative to the LPS-only model group, although small-to-medium effect sizes in the protective direction were observed for several measures, and BME demonstrated limited effects on classical inflammatory markers but exerted more pronounced modulation of prostaglandin signaling. The most consistent positive findings of the present study are therefore the phytochemical and antioxidant characterization of this standardized extract; the in vivo biochemical markers data support a model in which cholinergic and lipid mediator pathways are more responsive to therapeutic intervention than classical cytokine cascades under the present experimental conditions. Thus, the results should be regarded as preliminary directional evidence that warrants confirmation in larger-scale, dose-ranging studies.

## Data Availability

All data generated or analyzed during this study are included in this published article.

## Supplementary Materials

The following supporting information can be downloaded at: https://www.mdpi.com/article/10.3390/ijms27125229/s1.

## Abbreviations

The following abbreviations are used in this manuscript:

AAPH	2,2-azobis(2-amidinopropane) dihydrochloride
ABTS	2,2-azino-bis(3-ethylbenzothiazoline-6-sulfonic acid
AChE	Acetylcholinesterase
AD	Alzheimer’s disease
AlCl_3_	Aluminum chloride
ARRIVE	Animal research: Reporting of in vivo experiment
AUC	Area under the curve
BDNF	Brain-derived neurotrophic factor
BME	*Bacopa monnieri* extract
*C. elegeans*	*Caenorhabditis elegans*
CA1	Cornu ammonis-1
CA3	Cornu ammonis-3
CAE	Catechin equivalence
COX-2	Cyclooxygenase-2
DNA	Deoxyribonucleic acid
DPPH	2,2-diphenyl-l-picrylhydrazyl
*E. coli*	*Escherichia coli*
EDTA	Ethylenediaminetetraacetic acid
ELISA	Enzyme-linked immunosorbent assay
EP2	Prostaglandin E2 receptor subtype 2
EP4	Prostaglandin E2 receptor subtype 4
FeCl_3_	Ferric chloride
FeSO_4_	Ferrous sulfate
FeSO_4_.7H_2_O	Ferrous sulfate heptahydrate
FRAP	Ferric reducing antioxidant power
GAE	Gallic acid equivalence
HAT	Hydrogen atom transfer
HPLC	High-performance liquid chromatography
IACUC	Institutional Animal Care and Use Committee
IL-1β	Interleukin-1-beta
IL-6	Interleukin-6
iNOS	Inducible Nitric Oxide Synthase
K_2_S_2_O_8_	Potassium persulfate
LPS	lipopolysaccharide
MWM	Morris water maze
NaNO_2_	Sodium nitrite
NaOH	Sodium hydroxide
NBT	Nitroblue tetrazolium
NMDA	N-methyl-D-aspartate
NO	Nitric oxide
Nrf2	Nuclear factor erythroid 2-related factor 2
OD	Optical density
ORAC	Oxygen radical antioxidant capacity
PDA	Photodiode array detector
PGE_2_	Prostaglandin E2
RVS	Rivastigmine
SD	Standard deviation
SET	Single electron transfer
SPF	Specific pathogen free
TFC	Total flavonoid content
TLR4	Toll-like receptor-4
TNF-α	Tumor necrosis factor-alpha
TNFR2	Tumor necrosis factor receptor 2
TPC	Total phenolic content
TPTZ	2,4,6-tripyridyl-S-triazine hydrate
