# Supplementary material for: Phytochemical Profile, Antioxidant Activity, and Neuroprotective Effects of Bacopa monnieri Extract in a Lipopolysaccharide-Induced Dementia Model
Source: Int J Mol Sci. 2026 Jun 9;27(12):5229. doi: 10.3390/ijms27125229 (PMC13299668; doi:10.3390/ijms27125229)
Supplement: Supplementary file 1 [file ijms-27-05229-s001.zip › ijms-4290934-supplementary.pdf]

Phytochemical Profile, Antioxidant Activity, and Neuroprotective Effects of *Bacopa monnieri* Extract in a Lipopolysaccharide-Induced Dementia LPS

Supplementary File

Supplementary Table S1: The total phenolic and total flavonoid contents and antioxidant capacities of *Bacopa monnieri* extract

**Methods**	**Antioxidant capacity**
TPC	9.27±0.08 mg GAE/g extract
TFC	8.04±0.15 mg CAE/g extract
MCA	N/A
DPPH	3.05±0.07 (IC_{50}, mg/mL)
ABTS	1.95±0.04 (IC_{50}, mg/mL)
FRAP	7.83±0.01 µM $FeSO_4$/mg extract
NBT	0.59±0.01 IC_{50} mg/mL
ORAC	1.17±0.01 µM TE/mg extract

Note: Metal chelating activity (MCA), Free radical scavenging activities of DPPH and ABTS, Ferric reducing antioxidant power (FRAP), Nitroblue tetrazolium (NBT), Oxygen radical antioxidant capacity (ORAC), Not available (N/A).

Supplementary Table S2: Phytochemical compounds identified in *Bacopa monnieri* extract using HPLC analysis

Compounds	Equation	R^2	Retention time (min)	Peak area	Content (μg/mg extract)
Bacoside A_3	y = 794.21x + 5729.1	1.00	10.56±0.03	32675.33±136.95	33.93±0.17
Bacopaside II	y = 2133.7x + 7204.9	1.00	11.37±0.03	99054.00±603.03	43.05±0.28
Bacopaside X	y = 1456.3x + 8350.6	1.00	12.31±0.03	33749.00±479.70	17.44±0.33
Bacopasaponin C	y = 1645.1x - 4583.3	1.00	13.52±0.04	82136.00±106.04	52.71±0.06

Supplementary Table S3a: Results of absorbance using the food clearance assay in *C. elegans*

Concentration (mg/mL)	Day 1	Day 2	Day 3	Day 4	Day 5	Day 6	Day 7
Control	0.57±0.01	0.78±0.02	0.60±0.13	0.47±0.01	0.21±0.01	0.21±0.01	0.24±0.01
0.10	0.42±0.02*	0.71±0.03*	0.69±0.05*	0.65±0.01*	0.65±0.04*	0.74±0.04*	0.71±0.05*
0.25	0.42±0.01*	0.65±0.01*	0.61±0.03	0.58±0.01*	0.58±0.02*	0.65±0.04*	0.59±0.02*
0.50	0.49±0.02*	0.73±0.02*	0.73±0.06*	0.66±0.10*	0.11±0.01*	0.11±0.01*	0.09±0.02*
1.00	0.52±0.02*	0.75±0.02	0.74±0.08*	0.57±0.04*	0.11±0.01*	0.11±0.00*	0.09±0.00*

Note: The statistical differences compared with the control group $^*p<0.05$

Supplementary Table S3b: Results of feeding rate using pharyngeal pumping assay in *C. elegans*

**Concentration (mg/mL)**	**Pharyngeal pumping rate (pumps/min)**
Control	88.00±2.00
0.1	84.00±0.00
0.25	77.33±4.16*
0.5	68.67±1.15*
1.0	57.33±5.03*

Note: The statistical differences compared with the control group *$p<0.05$

Supplementary Table S4a: Results of escape latency at day 1-4 using the Morris water maze test

Group	Day 1 (sec)	Day 2 (sec)	Day 3 (sec)	Day 4 (sec)
Normal	43.98±2.76	18.64±2.19	19.50±2.04	13.91±2.76
LPS	43.14±3.34	27.40±2.05	16.58±2.85	17.35±2.94
RVS	28.62±2.64$^{*\#}$	15.45±1.83$^{\#}$	13.80±1.38	6.88±0.87$^{\#}$
BME	39.87±3.99	28.66±4.49*	17.94±3.60	10.93±1.38

Note: The statistical differences compared with the normal group $^{*}p<0.05$; compared with the LPS group $^{\#}p<0.05$. LPS- lipopolysaccharide, RVS- Rivastigmine, BME- *Bacopa monnieri* extract

Supplementary Table S4b: Results of path length at day 1-4 using the Morris water maze test

Group	Day 1 (cm)	Day 2 (cm)	Day 3 (cm)	Day 4 (cm)
Normal	1149.77±57.00	490.08±45.63	465.87±50.83	270.88±46.26
LPS	1075.05±74.70	615.14±62.94	422.08±60.21	400.21±62.42
RVS	669.75±48.42$^{*\#}$	374.91±41.83$^{\#}$	312.74±30.20	148.80±14.06$^{\#}$
BME	909.53±100.74*	537.63±63.95	448.54±72.91	277.57±39.35

Note: The statistical differences compared with the normal group $^{*}p<0.05$; compared with the LPS group $^{\#}p<0.05$. LPS- lipopolysaccharide, RVS- Rivastigmine, BME- *Bacopa monnieri* extract

Supplementary Table S5a: Results of behavioral assessment using the Morris water maze test

Group	Path length in target quadrant (cm)	Time spent in target quadrant (sec)	Number of crossing platform
Normal	485.96±38.58	27.72±1.71	2.25±0.31
LPS	391.24±36.39	18.54±1.77*	1.19±0.23*
RVS	326.89±35.27	19.10±1.13*	1.13±0.28*
BME	423.37±22.93	22.86±1.32*	1.00±0.15*

Note: The statistical differences compared with the normal group $^{*}p<0.05$. LPS- lipopolysaccharide, RVS- Rivastigmine, BME- *Bacopa monnieri* extract

Supplementary Table S5b: Results of behavioral assessment using the Y- maze test

Group	Path length in arm C (cm)	Time spent in arm C (sec)	Number of entries in arm C	%Alternation
Normal	1639.30±156.46	157.84±20.25	4.00±0.62	54.36±4.88
LPS	829.42±112.22*	72.29±15.63*	2.43±0.53	69.46±8.53
RVS	990.68±176.75*	145.47±23.62#	3.00±0.54	57.14±9.52
BME	1011.61±206.49*	77.40±18.75*	3.14±0.74	60.38±6.05

Note: The statistical differences compared with the normal group $^{*}p<0.05$; compared with the LPS group $^{\#}p<0.05$. LPS- lipopolysaccharide, RVS- Rivastigmine, BME- *Bacopa monnieri* extract

Supplementary Table S6a: Statistical analysis of behavioral data-omnibus ANOVA results

Test	Outcome	Effect	Test type	F	df1	df2	p-value	Partial η²	Significance
MWM training (Days 1–4)	Escape latency (s)	Group (between)	Mixed (split-plot) ANOVA	5.811	3	24	0.004	0.421	**
MWM training (Days 1–4)	Escape latency (s)	Day (within)	Mixed ANOVA, Greenhouse–Geisser	58.607	3	72	<0.001	0.709	***
MWM training (Days 1–4)	Escape latency (s)	Group × Day	Mixed ANOVA	1.497	9	72	0.166	0.158	ns
MWM training (Days 1–4)	Path length (cm)	Group (between)	Mixed (split-plot) ANOVA	9.320	3	24	<0.001	0.538	***
MWM training (Days 1–4)	Path length (cm)	Day (within)	Mixed ANOVA, Greenhouse–Geisser	85.917	3	72	<0.001	0.782	***
MWM training (Days 1–4)	Path length (cm)	Group × Day	Mixed ANOVA	1.822	9	72	0.079	0.185	ns
MWM probe trial (Day 5)	Distance in target quadrant (cm)	Group	One-way ANOVA	2.138	3	36	0.112	0.151	ns
MWM probe trial (Day 5)	Time in target quadrant (s)	Group	One-way ANOVA	2.750	3	35	0.057	0.191	ns
MWM probe trial (Day 5)	Platform crossings	Group	One-way ANOVA	6.129	3	35	0.002	0.344	**
Y-maze	Distance in novel arm (cm)	Group	One-way ANOVA	4.385	3	32	0.011	0.291	*
Y-maze	Time in novel arm (s)	Group	One-way ANOVA	5.833	3	33	0.003	0.347	**
Y-maze	Entries into novel arm	Group	One-way ANOVA	1.586	3	34	0.211	0.123	ns

Significance: ***p<0.001, **p<0.01, *p<0.05, ns = not significant. Partial η² (np2): small ≈0.01, medium ≈0.06, large ≈0.14. For the Day (within subject) effect, the Greenhouse–Geisser corrected p-value is reported. Significant p-values (<0.05) are highlighted. MWM- Morris Water Maze

Supplementary Table S6b: Mauchly's test of sphericity (within-subject factor: Day) and Levene's test for homogeneity of variance (between-subject factor: Group)

Outcome	Test	Statistic	χ^2	df	p-value	Assumption met
MWM Escape latency (Days 1–4)	Mauchly's W	0.7256	8.2489	5	0.143	Yes — sphericity assumed
MWM Path length (Days 1–4)	Mauchly's W	0.6954	9.3430	5	0.096	Yes — sphericity assumed
MWM probe: Distance in target quadrant (cm)	Levene's W	0.2277	—	—	0.877	Yes — variances homogeneous
MWM probe: Time in target quadrant (s)	Levene's W	0.1592	—	—	0.923	Yes — variances homogeneous
MWM probe: Platform crossings	Levene's W	2.1985	—	—	0.106	Yes — variances homogeneous
Y-maze: Distance in novel arm (cm)	Levene's W	0.6435	—	—	0.593	Yes — variances homogeneous
Y-maze: Time in novel arm (s)	Levene's W	0.6128	—	—	0.612	Yes — variances homogeneous
Y-maze: Entries into novel arm	Levene's W	0.2423	—	—	0.866	Yes — variances homogeneous

Note: MWM- Morris Water Maze

Supplementary Table S6c: Pairwise post-hoc comparisons (Tukey HSD), key contrasts only (Normal vs LPS, LPS vs Rivastigmine, LPS vs BME)

Test	Outcome	Day	Comparison	Mean diff (A–B)	SE	t	p-Tukey	Hedges' g	Significance
MWM training	Escape latency (s)	1	Normal vs LPS	1.831	5.163	0.355	0.984	0.190	ns
MWM training	Escape latency (s)	1	LPS vs RVS	10.479	5.163	2.030	0.205	1.042	ns
MWM training	Escape latency (s)	1	LPS vs BME	-0.474	5.163	-0.092	1.000	-0.040	ns
MWM training	Escape latency (s)	2	Normal vs LPS	-9.534	4.798	-1.987	0.221	-1.520	ns
MWM training	Escape latency (s)	2	LPS vs RVS	11.182	4.798	2.331	0.119	1.994	ns
MWM training	Escape latency (s)	2	LPS vs BME	-2.945	4.798	-0.614	0.927	-0.243	ns
MWM training	Escape latency (s)	3	Normal vs LPS	3.386	3.885	0.871	0.819	0.442	ns
MWM training	Escape latency (s)	3	LPS vs RVS	2.217	3.885	0.571	0.940	0.307	ns
MWM training	Escape latency (s)	3	LPS vs BME	-1.822	3.885	-0.469	0.965	-0.188	ns
MWM training	Escape latency (s)	4	Normal vs LPS	-1.528	3.513	-0.435	0.972	-0.163	ns
MWM training	Escape latency (s)	4	LPS vs RVS	9.486	3.513	2.700	0.057	1.432	ns
MWM training	Escape latency (s)	4	LPS vs BME	5.435	3.513	1.547	0.427	0.781	ns
MWM training	Path length (cm)	1	Normal vs LPS	67.714	123.960	0.546	0.947	0.321	ns
MWM training	Path length (cm)	1	LPS vs RVS	403.247	127.699	3.158	0.021	1.938	*
MWM training	Path length (cm)	1	LPS vs BME	164.313	127.699	1.287	0.580	0.519	ns
MWM training	Path length (cm)	2	Normal vs LPS	-160.472	91.970	-1.745	0.324	-0.892	ns
MWM training	Path length (cm)	2	LPS vs RVS	239.508	94.744	2.528	0.081	1.440	ns
MWM training	Path length (cm)	2	LPS vs BME	110.242	94.744	1.164	0.655	0.485	ns
MWM training	Path length (cm)	3	Normal vs LPS	47.007	86.902	0.541	0.948	0.259	ns
MWM training	Path length (cm)	3	LPS vs RVS	106.016	89.523	1.184	0.642	0.650	ns
MWM training	Path length (cm)	3	LPS vs BME	-29.670	89.523	-0.331	0.987	-0.140	ns

Test	Outcome	Day	Comparison	Mean diff (A–B)	SE	t	p-Tukey	Hedges' g	Significance
MWM training	Path length (cm)	4	Normal vs LPS	-129.332	63.266	-2.044	0.200	-0.862	ns
MWM training	Path length (cm)	4	LPS vs RVS	251.413	65.174	3.858	0.004	2.192	**
MWM training	Path length (cm)	4	LPS vs BME	116.103	65.174	1.781	0.306	0.799	ns
MWM probe (Day 5)	Distance in target quadrant (cm)		Normal vs LPS	89.809	59.925	1.499	0.449	0.603	ns
MWM probe (Day 5)	Distance in target quadrant (cm)		LPS vs RVS	54.292	59.925	0.906	0.802	0.428	ns
MWM probe (Day 5)	Distance in target quadrant (cm)		LPS vs BME	-32.672	58.620	-0.557	0.944	-0.249	ns
MWM probe (Day 5)	Time in target quadrant (s)		Normal vs LPS	7.953	3.180	2.501	0.077	1.021	ns
MWM probe (Day 5)	Time in target quadrant (s)		LPS vs RVS	-0.123	3.180	-0.039	1.000	-0.019	ns
MWM probe (Day 5)	Time in target quadrant (s)		LPS vs BME	-3.949	3.024	-1.306	0.565	-0.588	ns
MWM probe (Day 5)	Platform crossings		Normal vs LPS	0.980	0.304	3.221	0.014	1.234	*
MWM probe (Day 5)	Platform crossings		LPS vs RVS	0.042	0.304	0.139	0.999	0.062	ns
MWM probe (Day 5)	Platform crossings		LPS vs BME	0.200	0.312	0.639	0.919	0.369	ns
Y-maze	Distance in novel arm (cm)		Normal vs LPS	732.901	215.420	3.402	0.009	1.575	**
Y-maze	Distance in novel arm (cm)		LPS vs RVS	-162.317	215.420	-0.753	0.874	-0.405	ns

Test	Outcome	Day	Comparison	Mean diff (A–B)	SE	t	p-Tukey	Hedges' g	Significance
Y-maze	Distance in novel arm (cm)		LPS vs BME	-189.666	215.420	-0.880	0.815	-0.435	ns
Y-maze	Time in novel arm (s)		Normal vs LPS	80.980	24.462	3.310	0.012	1.480	*
Y-maze	Time in novel arm (s)		LPS vs RVS	-68.216	25.581	-2.667	0.054	-1.288	ns
Y-maze	Time in novel arm (s)		LPS vs BME	-6.272	25.581	-0.245	0.995	-0.149	ns
Y-maze	Entries into novel arm		Normal vs LPS	1.492	0.703	2.124	0.166	0.906	ns
Y-maze	Entries into novel arm		LPS vs RVS	-0.510	0.737	-0.692	0.900	-0.374	ns
Y-maze	Entries into novel arm		LPS vs BME	-0.697	0.737	-0.945	0.781	-0.438	ns

The mean difference is reported as A − B (positive value indicates A > B). Hedges' g is the small-sample-corrected Cohen's d effect size: |g| ≈ 0.2 small, 0.5 medium, 0.8 large. Significant p-values (p<0.05) are highlighted. LPS- lipopolysaccharide, RVS- Rivastigmine, BME- *Bacopa monnieri* extract, MWM- Morris Water Maze

Supplementary Table S6d: Descriptive statistics (mean ± SD, N) by group and outcome

Outcome	Group	N	Mean	SD
MWM Escape latency (s) — Day 1	Normal	7	42.764	7.576
MWM Escape latency (s) — Day 1	LPS	7	40.933	10.233
MWM Escape latency (s) — Day 1	RVS	7	30.454	8.526
MWM Escape latency (s) — Day 1	BME	7	41.407	11.762
MWM Escape latency (s) — Day 2	Normal	7	17.870	6.284
MWM Escape latency (s) — Day 2	LPS	7	27.404	5.426
MWM Escape latency (s) — Day 2	RVS	7	16.223	5.066
MWM Escape latency (s) — Day 2	BME	7	30.349	15.088
MWM Escape latency (s) — Day 3	Normal	7	19.499	5.393
MWM Escape latency (s) — Day 3	LPS	7	16.113	8.588
MWM Escape latency (s) — Day 3	RVS	7	13.896	4.192
MWM Escape latency (s) — Day 3	BME	7	17.935	9.535
MWM Escape latency (s) — Day 4	Normal	7	14.839	9.083
MWM Escape latency (s) — Day 4	LPS	7	16.367	8.462
MWM Escape latency (s) — Day 4	RVS	7	6.881	2.310
MWM Escape latency (s) — Day 4	BME	7	10.933	3.655
MWM Path length (cm) — Day 1	Normal	8	1149.772	161.226
MWM Path length (cm) — Day 1	LPS	6	1082.058	238.847
MWM Path length (cm) — Day 1	RVS	7	678.811	145.315
MWM Path length (cm) — Day 1	BME	7	917.745	334.296
MWM Path length (cm) — Day 2	Normal	8	478.766	141.761
MWM Path length (cm) — Day 2	LPS	6	639.238	199.869
MWM Path length (cm) — Day 2	RVS	7	399.730	102.863

Outcome	Group	N	Mean	SD
MWM Path length (cm) — Day 2	BME	7	528.996	220.649
MWM Path length (cm) — Day 3	Normal	8	465.875	143.761
MWM Path length (cm) — Day 3	LPS	6	418.868	201.207
MWM Path length (cm) — Day 3	RVS	7	312.852	92.256
MWM Path length (cm) — Day 3	BME	7	448.538	192.901
MWM Path length (cm) — Day 4	Normal	8	270.882	130.846
MWM Path length (cm) — Day 4	LPS	6	400.214	152.902
MWM Path length (cm) — Day 4	RVS	7	148.801	37.202
MWM Path length (cm) — Day 4	BME	7	284.112	118.540
MWM probe: Distance in target quadrant (cm)	Normal	10	448.276	153.173
MWM probe: Distance in target quadrant (cm)	LPS	9	358.467	128.915
MWM probe: Distance in target quadrant (cm)	RVS	10	304.176	113.569
MWM probe: Distance in target quadrant (cm)	BME	11	391.140	123.354
MWM probe: Time in target quadrant (s)	Normal	9	25.144	8.669
MWM probe: Time in target quadrant (s)	LPS	10	17.191	6.152
MWM probe: Time in target quadrant (s)	RVS	9	17.314	5.964
MWM probe: Time in target quadrant (s)	BME	11	21.140	6.709
MWM probe: Platform crossings	Normal	10	2.114	0.893
MWM probe: Platform crossings	LPS	10	1.134	0.599
MWM probe: Platform crossings	RVS	10	1.092	0.705
MWM probe: Platform crossings	BME	9	0.934	0.405
Y-maze: Distance in novel arm (cm)	Normal	9	1503.151	543.460
Y-maze: Distance in novel arm (cm)	LPS	9	770.250	312.451
Y-maze: Distance in novel arm (cm)	RVS	9	932.567	440.926
Y-maze: Distance in novel arm (cm)	BME	9	959.916	497.908

Outcome	Group	N	Mean	SD
Y-maze: Time in novel arm (s)	Normal	11	149.019	61.729
Y-maze: Time in novel arm (s)	LPS	8	68.039	34.516
Y-maze: Time in novel arm (s)	RVS	9	136.255	60.787
Y-maze: Time in novel arm (s)	BME	9	74.311	43.951
Y-maze: Entries into novel arm	Normal	11	3.806	1.792
Y-maze: Entries into novel arm	LPS	9	2.314	1.258
Y-maze: Entries into novel arm	RVS	9	2.824	1.334
Y-maze: Entries into novel arm	BME	9	3.011	1.736

LPS- lipopolysaccharide, RVS- Rivastigmine, BME- *Bacopa monnieri* extract, MWM- Morris Water Maze

Supplementary Table S7a: HPLC calibration parameters for the four bacoside reference standards used to quantify Bacopa monnieri extract constituents

Peak	Compound	Supplier	Catalog no.	Lot no.	Purity (%)	Calibration range (ppm)	Regression equation	R^2	LOD (ppm)	LOQ (ppm)
A	Bacoside A3	Sigma-Aldrich (Merck)	53889	BCCK7697	≥95%	12.5–1000.0	$y = 794.21 \cdot x + 5729.10$	0.9959	83.69	253.61
B	Bacopaside II	Sigma-Aldrich (Merck)	44698	BCCK7287	≥95%	12.5–1000.0	$y = 2133.67 \cdot x + 7204.86$	0.9975	65.77	199.29
C	Bacopaside X	Sigma-Aldrich (Merck)	PHL80365	134014970	≥95%	12.5–1000.0	$y = 1456.31 \cdot x + 8350.58$	0.9963	80.15	242.89
D	Bacopasaponin C	Sigma-Aldrich (Merck)	PHL80362	134014954	≥90.0%	12.5–1000.0	$y = 1645.07 \cdot x - 4583.33$	0.9994	31.32	94.91

Calibration was performed on a Waters Acquity Arc system with a photodiode array detector, monitored at 205 nm (processing method ProcessBacosideA_18072024, calibration ID 3398, calibrated 18 July 2024). Calibration concentrations: 12.5, 25, 50, 100, 250, 500, and 1000 ppm (μg/mL); 5 μL injection volume. Regression: linear ordinary least squares; y = peak area (arbitrary units), x = concentration (ppm). LOD = 3.3 × σ / slope; LOQ = 10 × σ / slope, where σ is the standard error of the regression residuals (n − 2 degrees of freedom).

Supplementary Table S7b: Alternative calibration parameters using working range only (50–1000 ppm), excluding the two lowest standards where deviation exceeded ±20% for three of the four compounds

Peak	Compound	Calibration range (ppm)	Regression equation	R^2	LOD (ppm)	LOQ (ppm)	n points
A	Bacoside A3	50.0–1000.0	$y = 784.74 \cdot x + 12491.89$	0.9952	102.99	312.09	5
B	Bacopaside II	50.0–1000.0	$y = 2116.50 \cdot x + 19471.52$	0.9969	81.92	248.24	5
C	Bacopaside X	50.0–1000.0	$y = 1440.77 \cdot x + 19445.62$	0.9955	99.28	300.86	5
D	Bacopasaponin C	50.0–1000.0	$y = 1641.85 \cdot x - 2276.99$	0.9993	40.00	121.22	5

Note: For three of the four compounds (peaks A, B, and C), the % deviation of back-calculated vs nominal concentration exceeded ±20% at the two lowest calibration points (12.5 and 25 ppm). The full 7-point curve was retained for the present analysis (reported in Supplementary Table 7a) because all sample concentrations of interest fell within the linear range of the upper five standards.

Supplementary Table S7c: Raw calibration data points (nominal concentration, peak area, back-calculated concentration, and % deviation) for each standard

Compound	Level	Nominal X (ppm)	Peak area	Back-calc. (ppm)	% Deviation
Bacoside A3	1	12.5	7768.43	2.568	-79.458
Bacoside A3	2	25	17658.64	15.021	-39.918
Bacoside A3	3	50	37149.05	39.561	-20.878
Bacoside A3	4	100	79502.18	92.888	-7.112
Bacoside A3	5	250	219252.68	268.850	+7.540
Bacoside A3	6	500	437332.75	543.436	+8.687
Bacoside A3	7	1000	780225.75	975.176	-2.482
Bacopaside II	1	12.5	20966.76	6.450	-48.401
Bacopaside II	2	25	44745.29	17.594	-29.623
Bacopaside II	3	50	95131.83	41.209	-17.581
Bacopaside II	4	100	203674.25	92.081	-7.919
Bacopaside II	5	250	574315.64	265.791	+6.317
Bacopaside II	6	500	1145860.15	533.661	+6.732
Bacopaside II	7	1000	2099724.62	980.714	-1.929
Bacopaside X	1	12.5	14457.28	4.193	-66.454
Bacopaside X	2	25	30895.13	15.481	-38.077
Bacopaside X	3	50	65332.22	39.127	-21.745
Bacopaside X	4	100	141925.78	91.722	-8.278
Bacopaside X	5	250	401466.49	269.940	+7.976
Bacopaside X	6	500	795654.60	540.617	+8.123
Bacopaside X	7	1000	1430318.00	976.420	-2.358
Bacopasaponin C	1	12.5	14635.27	11.683	-6.540
Bacopasaponin C	2	25	32470.13	22.524	-9.904
Bacopasaponin C	3	50	69556.47	45.068	-9.864
Bacopasaponin C	4	100	148939.89	93.323	-6.677

Compound	Level	Nominal X (ppm)	Peak area	Back-calc. (ppm)	% Deviation
Bacopasaponin C	5	250	420685.93	258.511	+3.404
Bacopasaponin C	6	500	842737.21	515.066	+3.013
Bacopasaponin C	7	1000	1626218.87	991.326	-0.867

Yellow-highlighted % deviation values indicate calibration points where back-calculated concentration deviates from nominal by more than ±20%. Per the Empower-3 calibration report, none of these points were marked as 'Manual' or 'Ignore' during processing; they remain in the calibration curve as reported in Supplementary Table 7a.

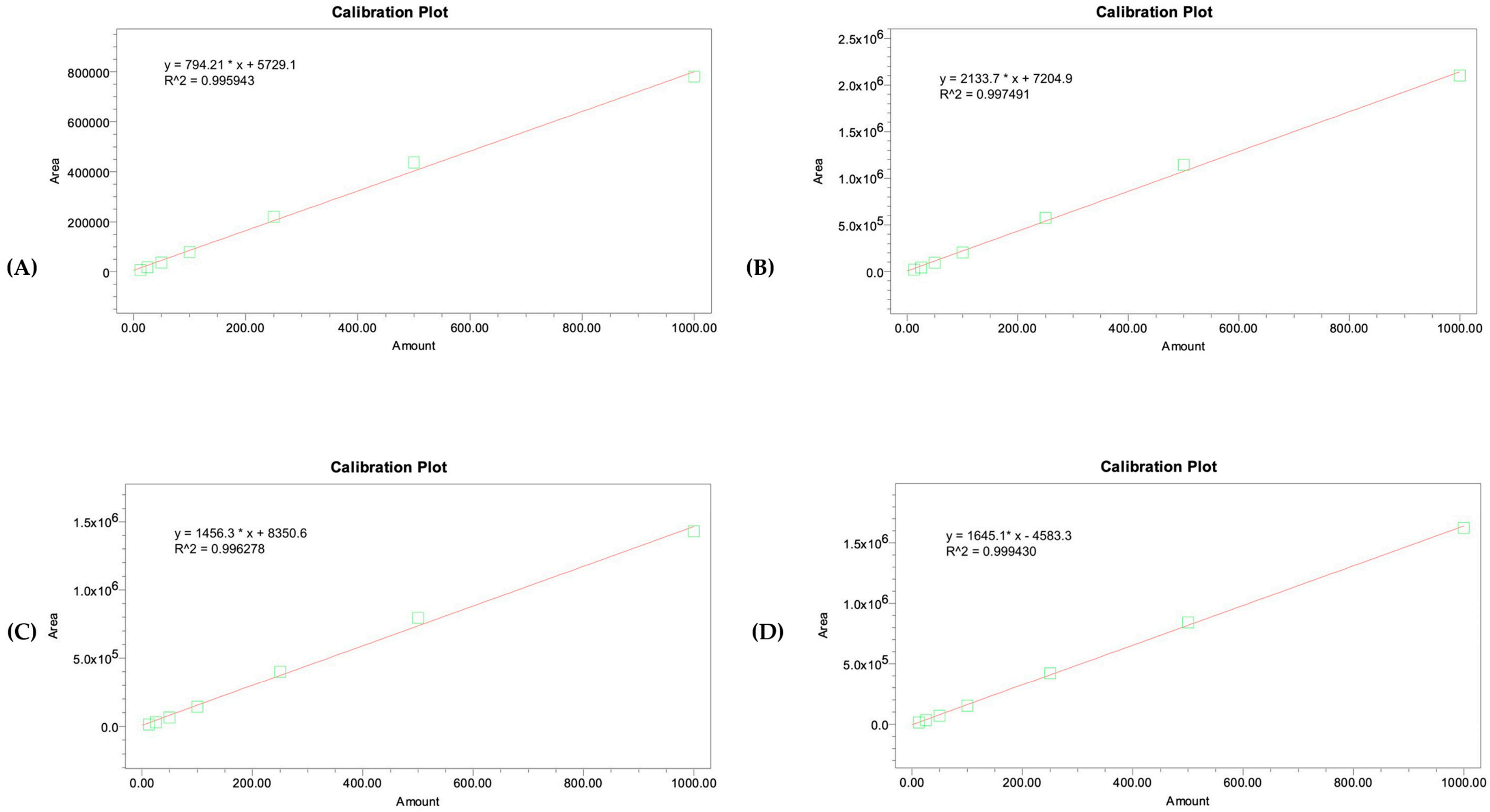

Supplementary Figure S1. Standard curve of four (4) components of bacoside A standard. (A) bacoside A3, (B) bacopaside II, (C) bacopaside X, and (D) bacopasaponin C
